# Engaging Biomedical Engineering in Health Disparities Challenges

**DOI:** 10.4172/2161-0711.1000595

**Published:** 2018-03-05

**Authors:** Maribel Vazquez

**Affiliations:** Department of Biomedical Engineering, City College of New York, USA

**Keywords:** Health disparities, Ethics, Undergraduate education, Societal impact

## Abstract

Health Disparities (HD) are community-based, biomedical challenges in need of innovative contributions from Science, Technology, Engineering and Math (STEM) fields. Surprisingly, STEM professionals demonstrate a persistent lack of HD awareness and/or engagement in both research and educational activities. This project introduced Health Disparities (HD) as technical challenges to incoming undergraduates in order to elevate engineering awareness of HD. The objective was to advance STEM-based, HD literacy and outreach to young cohorts of engineers. Engineering students were introduced to HD challenges in technical and societal contexts as part of Engineering 101 courses. Findings demonstrate that student comprehension of HD challenges increased via joint study of rising health care costs, engineering ethics and growth of biomedical-related engineering areas.

## Introduction

Health Disparities (HD) are preventable differences in the incidence, prevalence, mortality and burden of disease on communities targeted by factors such as gender, residence, ethnicity and/or socioeconomic status [1]. HD have become contemporary biomedical challenges with adverse and escalating effects in the United States (US) and worldwide. The cost of US health care has risen dramatically in the current decade, which has, both, aggravated federal spending forecasts and highlighted a charged political climate surrounding health inequality [2]. The portion of increased costs attributed to HD has yet to be quantitatively and objectively evaluated, but is certain to rise commensurate with currently-reported levels of HD across different communities [3,4].

The interdisciplinary nature of engineering can help decipher many current and developing HD challenges by leveraging engineering underpinnings to integrate technology with fundamental science, clinical therapies and health outcomes. Further, joint biomedical- related engineering ventures have impacted US public policy [5], health initiatives [6] and community-based challenges [7], all of which can uniquely address HD challenges. In order to fully evaluate HD as technical engineering challenges, however, the community must address the persistent lack of HD engagement among professionals in Science, Technology, Engineering, and Math (STEM) disciplines. Researchers and educators must also ameliorate the lack of HD awareness among the youngest engineers-in-training, e.g. PK-12 and undergraduates [8,9].

This brief describes a 4-year project undertaken to advance STEM- based HD literacy [10] and outreach to current and future STEM professionals. The objective was to elevate engineering awareness of HD challenges by incorporating health care data alongside ongoing HD research into introductory courses that describe potential career paths and research directions for engineers.

## Materials and Methods

This project was conducted with incoming undergraduate cohorts (first year students and transfers) at the Grove School of Engineering at the City College of New York (CCNY) over 4 years. Students in the introductory engineering course (ENGR 101) were given surveys and assignments to complete, whose results were statistically analyzed using the student’s t-test and post-hoc Tukey test. The overall course goal was to expose new students to potential engineering careers using small projects and assignments that cultivate their requisite technical skills. Each cohort in this study was comprised of 135–155 students, per year, all of whom were declared engineering majors and were predominantly under the age of 25.

CCNY heralds the only accredited, public engineering school in New York City, and is the flagship campus of the 24 schools that comprise the City University of New York (CUNY). CCNY is also a Minority Serving Institution [11] in which more than 51% of enrolled students identify as African-American, Hispanic-American, Native- American or Pacific Islander (as per guidelines of the US National Science Foundation, NSF).

## Results

This project introduced the concepts of health disparities (HD) to incoming students via the required, introductory course in Engineering. This was executed in two parts: (a) Placing HD and STEM fields in the context of US health care costs and (b) Using HD data as part of technical engineering training. Assessment of increased student HD awareness upon course completion was also measured.

### Rising health care costs and opportunities for engineers

**Figure 1:**
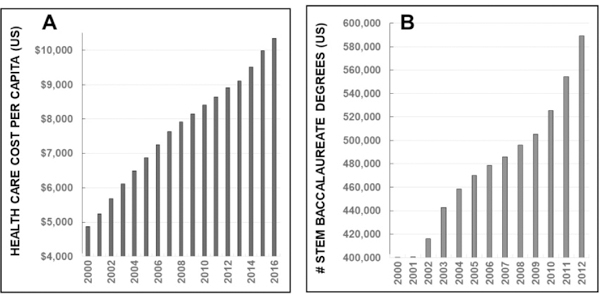
Current trends in US health care costs and STEM education. (A) The rising cost of US health care per capita over time (Source CMS.gov); (B) Increasing numbers of US accredited baccalaureate degrees in STEM fields awarded per year (source NSF.gov).

Incoming engineering students were introduced to the rising costs of US health care alongside the increasing numbers of STEM majors. As shown in Figure 1A, the federal expense of health care per capita currently approaches $10,500 or 18% of the country’s Gross Domestic Product [12] (GDP, per the US Center for Medicare and Medicaid Services www.cms.gov). More poignantly, this costly trend has increased dramatically in the current decade and is expected to continue. In complement, data in Figure 1B illustrates the growing numbers of STEM baccalaureate degrees awarded from US accredited programs [13], as per NSF analyses (www.nsf.gov). This academic trend has steadily increased through 2017, where nearly one third of all US baccalaureate degrees were awarded in STEM [14].

**Figure 2:**
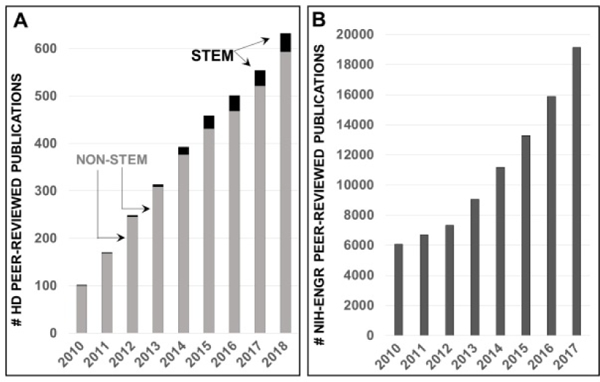
Growth of US federally-funded Health Disparities research alongside engineering research. (A) Growth of peer-reviewed research publications in Health Disparities funded by the National Institutes of Health (NIH) in the current decade; both STEM and non-STEM-based studies depicted; (B) Rising numbers of NIH-funded peer-reviewed publications from engineering groups (ENGR).

Using this national data, courses discussed unique interdisciplinary opportunities for engineers in health care and policy, as well as introduced the concepts of HD and community-based challenges in global and US health. Figure 2A illustrates the rising numbers of peer-reviewed HD research articles with funding from the US National Institutes of Health (NIH) alongside the small but growing fraction of STEM-based HD contributions since 2010. By contrast, Figure 2B depicts the exponential rise in engineering, peer-reviewed publications with NIH funding in the present decade. These data side-by-side illustrate the need and opportunities for engineers to address HD challenges within their developing careers.

### Integration of health disparities into engineering preparation

The second part of the project utilized HD data for technical assignments that developed statistical problem-solving skills needed in engineering professions. Students manipulated and analyzed epidemiological data from select US case studies of well-known HD challenges, e.g. cardiovascular disease [15], obesity [16] and vision loss [17]. In complement, engineers examined peer-reviewed literature of well-studied origins and agents of HD (per case study) as part of engineering ethics. Here, students were introduced to the research code of conduct [18] and its overlap with themes in social science [19], community health and public policy [20]. As per Table 1, the material highlighted how each HD agent related to STEM research and clinical trials [21–26], genetic screening [27–29], engineering technology [30,31], health care policy/access [32,33], quality of care [34–38] and historical societal factors [39–44].

### Assessment of increased health disparities awareness

**Figure 3:**
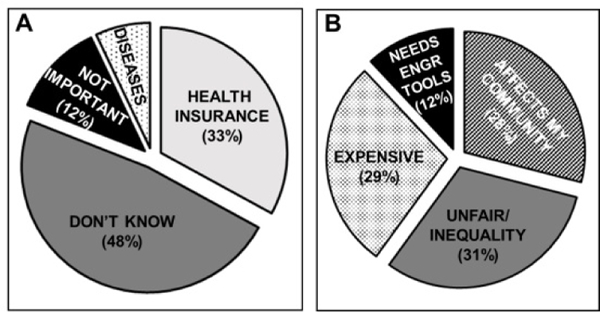
Student responses to ‘Why are challenges in US Health Disparities important? (A) Mean answers at the beginning of the course and (B) at course completion. Data reported is the mean value +/− one deviation of 4 years of incoming engineering cohorts.

The project, lastly, measured student awareness of HD challenges and how BME could be expanded with an HD context. Students were asked to provide an answer to the question, ‘Why are challenges in US Health Disparities important?’ at both the beginning and end of the course. As shown in Figure 3A, on the first day, 45% of students answered ‘I don’t know’, while 33% listed different types of health insurance plans. Another 12% claimed HD challenges were not important and the remaining students stated that HD was only relevant with certain diseases (notably diabetes was specifically mentioned in nearly all cases [45]).

By contrast, Figure 3B illustrates the definitions provided to the fill- in question at course completion. As seen, the majority of students stated HD challenges were important because they were caused by, or were symptoms of, inequity and unfairness in health services, health research, and/or health care (31%). Other large student cohorts replied that HD challenges were important because ‘They disproportionately affect my community’ (28%) and were ‘A great national expense’ (29%).Another 12% of undergraduates stated that HD challenges were important because they were in need of engineering tools.

## Discussion

The growth of Health Disparities (HD) across different communities of Americans represents a biomedical challenge that has engaged few engineering professionals. Technical curricula lag in introducing HD challenges as opportunities for engineering innovation and impact, despite record numbers of STEM degrees awarded this decade. This brief describes a strategy with which to introduce engineering majors to HD challenges in a technical context. Introductory 101 courses became ideal nucleation sites because HD comprehension requires a technical grasp of statistical analyses fundamental to all engineering experiments. In addition, HD challenges simultaneously introduce contemporary themes within engineering ethics that interface with public policy, generational societal activism [46] and ABET accreditation requirements [47]. As such, data sets that highlighted the incidence or progression of disease within communities targeted by residence, age and/or education level (for example) elucidated HD in joint technical and societal contexts. Such a juxtaposition is often cited for selection of the engineering major (particular biomedical engineering [47]), underscoring HD challenges as natural, but underdeveloped, areas for engineering innovation.

The responses from engineering cohorts recorded in this project illustrate two main themes. First, incoming engineers were often unaware of the high cost of health care or the prevalence of HD in the US. This is expected, as students can rely upon their parents for US medical insurance until the age of 25, as a whole are among the healthiest cohorts of Americans, and are minimally exposed to disparities in disease progression and/or burden within their age group [48]. Second, collective awareness of HD resonated most strongly through personal connections with communities adversely affected by HD challenges, as well as through the demonstrated imbalance in inflated cost and high inequality in American health. These themes are meaningful for engineering engagement in HD, as students who question HD underpinnings are motivated to use their engineering skills to help achieve medical parity. Further, associating HD with one’s own communities will produce a larger diversity of professionals who undertake HD challenges, uplifting US workforce diversity in STEM and overall [40,43,49]. In these ways, early exposure to HD challenges may help engineers continue in HD-related careers via advanced study in graduate, medical or law schools, as well as increasingly quantitative programs in community health, physician assistant [50] or advanced nursing specialties [51].

Future projects will develop comprehensive strategies to increase engineering engagement in HD by: (a) Integrating HD data into research and engineering design projects; (b) Incorporating underlying HD medical principles into required STEM courses; and (c) Using HD challenges to improve experimental design procedures. Ambitious programmatic actions include developing an HD-based engineering ethics curriculum and creating an HD certificate program or approved engineering minor in Health Disparities jointly with community and public health programs.

## Conclusions

Introducing engineering undergraduates to HD literature in joint technical and societal contexts increases student awareness and comprehension of these complex community-based challenges. Early exposure to HD as engineering challenges will help increase the number of HD-related researchers in STEM and incorporate HD into engineering ethics and technical curricula.

## Figures and Tables

**Table 1:** Summary of peer-reviewed literature describing well-known agents of Health Disparities discussed in engineering alongside US case studies.

HD Agents	Description of Concerns	Citation
Clinical Trials	Homogeneous patient groups	[21,22,25,41]
	Gender imbalance	[23,24,26]
Genetic Screening	Lacking determinants of disease progression	[28,29]
	Non-inclusive of resistance to treatments/therapies	[37]
Technologies and Tools	Tested using homogenous patient groups	[27,30,31]
Access and Quality	Insurance cost and billing	[32,33,36]
	Physician and clinical networks	[34,35]
Historical and Societal Factors	Distrust of medical community	[38,39,42]
	Non-diverse workforce	[40,43,44]
